# Selection of Optimal Auxiliary Soil Nutrient Variables for Cokriging Interpolation

**DOI:** 10.1371/journal.pone.0099695

**Published:** 2014-06-13

**Authors:** Genxin Song, Jing Zhang, Ke Wang

**Affiliations:** Institute of Agricultural Remote Sensing and Information Technique, Zhejiang University, Hangzhou, Zhejiang, China; and Ministry of Education Key Laboratory of Environmental Remediation, Ecological and Health, Zhejiang University, Hangzhou, Zhejiang, China; Centro de Investigacion Cientifica y Educacion Superior de Ensenada, Mexico

## Abstract

In order to explore the selection of the best auxiliary variables (BAVs) when using the Cokriging method for soil attribute interpolation, this paper investigated the selection of BAVs from terrain parameters, soil trace elements, and soil nutrient attributes when applying Cokriging interpolation to soil nutrients (organic matter, total N, available P, and available K). In total, 670 soil samples were collected in Fuyang, and the nutrient and trace element attributes of the soil samples were determined. Based on the spatial autocorrelation of soil attributes, the Digital Elevation Model (DEM) data for Fuyang was combined to explore the coordinate relationship among terrain parameters, trace elements, and soil nutrient attributes. Variables with a high correlation to soil nutrient attributes were selected as BAVs for Cokriging interpolation of soil nutrients, and variables with poor correlation were selected as poor auxiliary variables (PAVs). The results of Cokriging interpolations using BAVs and PAVs were then compared. The results indicated that Cokriging interpolation with BAVs yielded more accurate results than Cokriging interpolation with PAVs (the mean absolute error of BAV interpolation results for organic matter, total N, available P, and available K were 0.020, 0.002, 7.616, and 12.4702, respectively, and the mean absolute error of PAV interpolation results were 0.052, 0.037, 15.619, and 0.037, respectively). The results indicated that Cokriging interpolation with BAVs can significantly improve the accuracy of Cokriging interpolation for soil nutrient attributes. This study provides meaningful guidance and reference for the selection of auxiliary parameters for the application of Cokriging interpolation to soil nutrient attributes.

## Introduction

The spatial distribution and variation of soil attributes are of considerable interest in soil science. A detailed understanding of the spatial variability of soil is the foundation for precision and variable agriculture management [1]. Soil attribute interpolation is critical for studying the spatial variation and distribution characteristics of soil. Analyzing and forecasting the spatial distribution and dynamics of soil properties are important elements of sustainable land management. In recent years, geostatistics has been widely used to predict the spatial distribution of physical and chemical soil properties [2]. Furthermore, Cokriging interpolation has been increasingly applied to all aspects of soil property prediction because it provides a higher level of prediction accuracy than ordinary kriging interpolation [3]. The Cokriging method, as an extension of the ordinary statistical kriging method, can obtain good results by allowing for more than one variable in a prediction and by considering both self-correlation and cross-correlation between variables [4]. Existing research has demonstrated that calculation of the cross-correlation using the Cokriging method can still achieve accurate prediction results even with a lack of simple correlation between variables [5], [6]. Chai Xurong et al. found that the Cokriging method can yield more accurate results than the ordinary kriging method when using auxiliary elevation as a parameter to predict the spatial distribution of soil exchangeable potassium and pH. Jiang Yong et al. compared the Cokriging method using zinc content (0–10 cm) in the upper soil as an auxiliary variable and the ordinary kriging method for predicting the distribution of zinc content (10–20 cm) in the lower soil, and they found that the Cokriging method yields more accurate results [7]. Liu Bo et al. used the Cokriging method to predict the spatial distribution of soil heavy metals in the city of Kunshan, and they found that use of the Cokriging method yields more accurate results compared to the ordinary kriging method for most heavy metal predictions [8].

The spatial distribution of one soil attribute is often closely related to the spatial distribution of other soil attributes, as all soil attributes are affected by the same regionalization phenomena or space process [9], [10]. With the rapid accumulation of data related to soil attributes from other sources, an increasing amount of data is applicable for use as auxiliary parameters for the Cokriging method. These optional auxiliary parameters can be divided into the following three categories: (1) data for various attributes obtained directly from soil samples; (2) soil type, topography, geomorphology, remote-sensing images, and soil spectral data, which are closely related to the collection of soil samples; and (3) influencing factors, which are related to human activities, including land utilization type as well as industrial, mining, and traffic layouts. The Cokriging method has become an effective tool for soil attribute interpolation. However, selection of the best parameters for Cokriging predictions from the many available auxiliary parameters has become a serious problem due to the lack of understanding of how to select the best auxiliary parameter from the rich related data as well as how to select the second best auxiliary parameter when data for the best auxiliary parameter are unavailable. In this paper, interpolation of soil nutrient elements (i.e., organic matter (OM), total N (TN), available P (AP), and available K (AK)) was used as an example to determine how to select the best auxiliary parameters for Cokriging interpolation. Other relevant data (i.e., soil attribute data and terrain data) were considered as auxiliary parameters. The example also demonstrated how second best data can be used to ensure the accuracy of Cokriging interpolation when the best auxiliary parameters are insufficient.

## Materials and Methods

### Ethics statement

This study was approved by the City Agricultural Office Department of Fuyang District, which monitors farmland nutrients. All of the data in this study can be published and shared.

### Study area

The study was conducted in the Fuyang District of Hangzhou, Zhejiang, China. The Fuyang District is located in northwestern Zhejiang Province, which lies between 45° 44′ 29″ to 30° 11′ 59″ N latitude and 119° 25′ 00″ to 120° 09′ 30″ W longitude. The geographical area of Fuyang is 1,821.10 km^2^, and the elevation ranges between 4 and 705 m. The terrain of Fuyang is tilted from southwest to northeast, and the central Fuchun River has an oblique penetration of 52 km. The soil is varied, fertile land with rich agricultural natural resources that are suitable for a variety of crops. The planting industry is also developed.

### Soil nutrient sampling data

**Figure 1:**
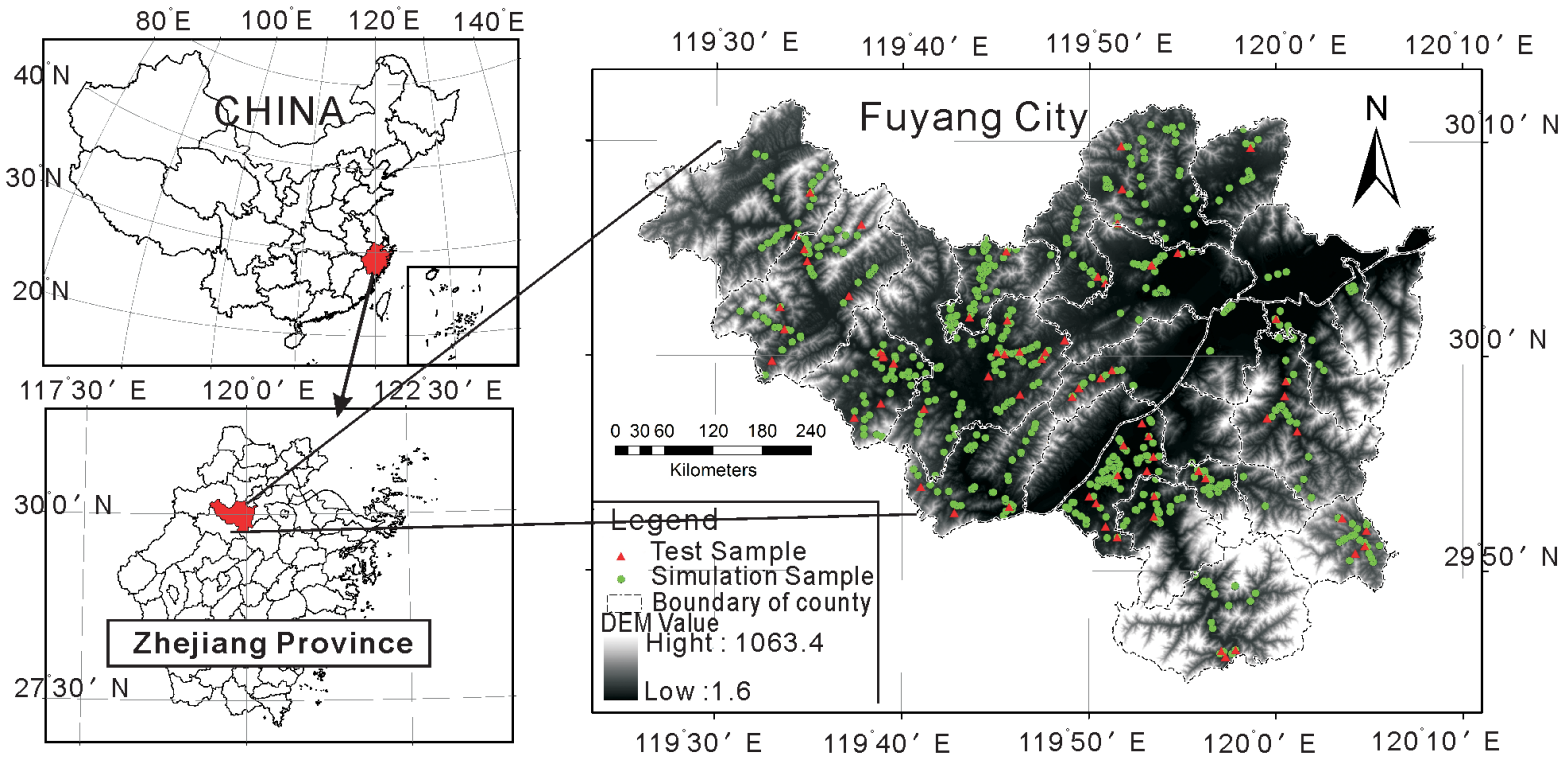
Distribution of sample points.

In this study area, the soil nutrient sampling data for this paper was the Fuyang soil monitoring data from 2006, and this data included 670 sample points. Each sample point included soil OM, TN, AP, and AK as well as a record of the latitude and longitude from GPS measurements. The sample point distribution is shown in Figure 1.

In this paper, the authors randomly divided 670 sampling points into simulation and test datasets with 600 and 70 points, respectively [11]. This study also simulated the spacing of soil nutrient properties using simulation datasets and evaluated the accuracy of the simulation using test sample datasets [12], [13].

### Soil trace element data

This article selected soil trace element data that were acquired at the same sample points as the soil nutrient sampling data. These data included laboratory samples tested for effective sulfur (ES), commutative hydrogen (CH), commutative aluminum (CA), commutative magnesium (CM), commutative calcium (CC), effective manganese (EMG), effective copper (EC), effective zinc (EZ), and effective molybdenum (EMO) [14]. In total, nine soil trace elements were considered.

### Terrain data

In this paper, the slope and aspect for Fuyang were created based on data from the 25-m resolution DEM for Fuyang and through digital terrain analysis technology to extract soil terrain information for sampling points. Extraction of the terrain index mainly included height (H), slope (β), and aspect (α). However, because the aspect information was from the perspective of the due north direction going clockwise, values ranged from 0 to 360°. Therefore, the aspect was changed to the sine and cosine values [15].

### Sampling point processing

Using Fuyang soil sampling point data provided by the agriculture department and according to the GPS coordinates of the sampling points, ArcGIS software was used to identify the sample point location on the Fuyang basic datum and to join the soil nutrient properties to each sample point. Based on the sample point test number, the nine soil trace element data points were then assigned to each sample point and to 670 sampling points. Points were randomly divided into the simulation and test datasets. The simulation dataset was used to interpolate the nutrient elements, and the test datasets were used to evaluate the accuracy of the interpolation results [16].

### Extraction and analysis of terrain attributes

The slope and aspect data generated from the 25-m resolution DEM for Fuyang and through digital terrain analysis technology to extract soil terrain information were used to determine the location of the sampling points. Extraction of the terrain index mainly included height (H), slope (β), and aspect (α). However, because the aspect expression was from the northern direction going clockwise, values ranged from 0 to 360°. Therefore, sine and cosine values were used in place of the aspect. Thus, the extracting terrain index included H, β, the sine value of the aspect (sinα), and the cosine value of the aspect (cosα). ArcGIS spatial analysis was used to extract terrain information for each sampling point location. Finally, the correlation analysis for soil OM, TN, AP, and AK as well as terrain parameters was performed using SPSS19 software [17].

### Correlation analysis for soil nutrient elements

There may be a certain degree of correlation for OM, TN, AP, and AK with the other elements, which has become a topic of interest for research scholars. In this paper, a correlation analysis was performed with SPSS19 software for OM, TN, AP, and AK to obtain the above four results of the correlation analysis among nutrient elements.

### Association correlation analysis between soil trace elements and soil nutrients

During the process of soil formation and development, a correlation exists between soil nutrient elements and soil trace elements. Using SPSS19 software, this study selected soil ES, EH, EA, EMG, ECA, EMA, ECO, EZ, and EMO (a total of nine soil trace elements) to perform a cross-correlation analysis with the four soil nutrient elements (OM, TN, AP, and AK).

### Cokriging interpolation method

Cokriging is a variation of the ordinary kriging method that is used when there is a close relationship between the spatial distributions of certain soil properties and other properties at the same position [18]. In particular, Cokriging is useful when it is difficult to obtain certain properties but not others. The Cokriging method is the best valuation method for regionalized variables from a single development to two or more coordinated regional attributes [19], and it uses a space correlation between two or more variables due to the autocorrelation of the main variables and cross-correlation of auxiliary variables. Estimates were made to improve the accuracy and rationality of the estimation.

The Cokriging prediction model can be summarized as follows:
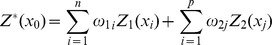
where 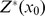 is the position of the sample point; 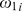 and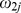 are two regionalized variables; and Z_1_ (X_i_) and Z_2_ (X_j_) are weight coefficients [20], [21], [22].

### Results obtained from the validation method

This article used test datasets, which contained 70 reserved sample points, to evaluate the accuracy of Cokriging interpolation results that used the best auxiliary variables (BAVs) and poor auxiliary variables (PAVs), and the Cokriging interpolation results were then compared to results from two predictions. Specifically, the BAVs and PAVs from the Cokriging interpolation results were assigned to the 70 test sample points to obtain simulation values for the two interpolation results. A comparative analysis was then performed on the two simulation values and measured values from the 70 test points. The contrast index mainly included correlation coefficients and mean absolute errors for the simulation values and measured values in item 2 [23].

## Results and Discussion

### Descriptive analysis of soil attributes

SPSS19 was used to perform statistical analyses for the soil nutrient elements for all points (OM, TN, AP, and AK). The results are presented in Table 1.

**Table 1:** Descriptive Statistics for Soil Nutrient Attributes.

	N	Mean (mg/kg)	Std. Deviation	Coefficient of Variation	Skewness	Kurtosis
OM (g/kg)	670	32.54	1.06	32.615	1.39	4.83
SN (g/kg)	670	1.946	0.06	31.579	0.67	0.81
AP (g/kg)	670	36.79	58.25	158.331	3.82	16.87
AK (g/kg)	670	90.18	72.63	80.539	2.94	10.95

Based on the coefficient of variation grading scale [24], OM and SN were classified as moderate variable. An analysis of the probability distribution of the original sampling point data indicated that the distribution of the nutrient attribute data from each sampling point exhibited clear deviations. Thus, the original data were logarithmically transformed and BOX-COX transformed so that the transformed data conformed to a normal distribution. The transformed data were used to interpolate the simulated nutrient attribute for each sampling point.

### Correlation analysis of terrain factors and soil attributes

ArcGIS was used to generate the slope and aspect map based on Fuyang 25-m resolution DEM data, and the corresponding terrain data were then extracted for each sampling point. SPSS19 was then used to calculate simple Pearson correlation coefficients for the four terrain factors and soil nutrient attributes as shown in Table 2. Soil OM, TN, and AK showed a significant relationship with elevation, slope, and sinα.

**Table 2:** Correlation Analyses of Soil Nutrient Attributes and Optional Auxiliary Parameters.

	OM	TN	AP	AK
OM	1	0.93**	0.30**	0.32**
TN	0.93**	1	0.18**	0.22**
AP	0.30**	0.18**	1	0.52**
AK	0.32**	0.22**	0.52**	1
H	0.11**	0.09*	0.03	0.05
β	0.03	−0.01	0.00	0.02
sinα	−0.03	0.01	−0.06	−0.09*
cosα	−0.09*	−0.08*	0.03	−0.01
ES	−0.01	−0.01	0.08	0.00
CH	0.11**	−0.03	0.35**	0.33**
CA	−0.07	−0.18**	0.05	0.23**
EMG	0.02	0.11**	0.02	0.03
CC	0.04	0.16**	−0.01	−0.07
EMA	0.16**	0.14**	0.28**	0.13**
EC	−0.06	−0.11**	0.01	0.00
EZ	0.08	0.08*	0.17**	0.16**
EMO	0.20**	0.22**	0.14**	0.11**

**Correlation is significant at the 0.01 level (two-tailed).

*Correlation is significant at the 0.05 level (two-tailed).

### Correlation analysis among attributes

SPSS19 was used to calculate simple Pearson correlation coefficients for nine trace elements with the four soil nutrient attributes. A correlation analysis was performed for the four soil nutrient attributes as shown in Table 2. The results indicated that there were significant correlations at the 0.01 level for the four soil nutrient attributes. The correlation between OM and TN was 0.932. Therefore, based on the relevant relationship among the four attributes, a certain attribute can be set as the target attribute to perform the Cokriging interpolation, and the other three soil nutrient attributes can be used as auxiliary parameters. Trace elements had a more significant correlation with nutrient attributes, especially for the correlation of TN, AK, and trace elements with EM, EC, EC, and EH.

### Optimizing the choice of auxiliary parameters

The above correlation analyses between soil attributes and terrain factors as well as among soil attributes demonstrated that each soil nutrient attribute had significant correlations with multiple terrain factors, trace elements, and other soil nutrient attributes [25]. Therefore, the terrain factors, trace elements, and other related soil nutrient attributes can be used as auxiliary parameters for a Cokriging interpolation of soil nutrient attributes [26]. The above correlation analyses indicated that there was a high correlation between auxiliary parameters and soil nutrient attributes. Furthermore, the correlations between other parameters and soil nutrients differed. In the process of performing the Cokriging interpolation, we can accord different degrees of correlation to these factors and select the most relevant auxiliary parameters [27].

### Interpolation results

**Figure 2:**
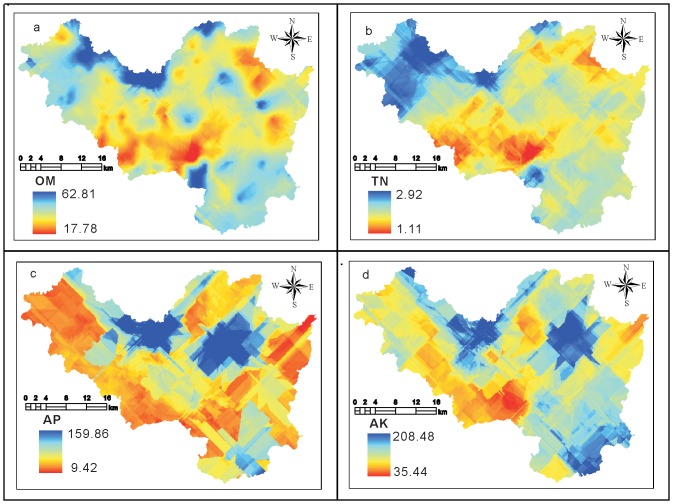
Cokriging prediction results using the BAVs.

Based on the previous results, OM, TN, AP, and AK were set as predicted targets for the Cokriging interpolation, and the three most significant correlation variables were selected as auxiliary variables [28]. TN, AP, and AK were set as auxiliary parameters for the interpolation of OM. OM, AP, and EMO were selected as auxiliary parameters for the interpolation of TN. AK, EH, and OM were selected as auxiliary parameters for the interpolation of AP. AP, EH, and OM were selected as auxiliary parameters for the interpolation of AK. The prediction results are presented in Figure 2.

**Figure 3:**
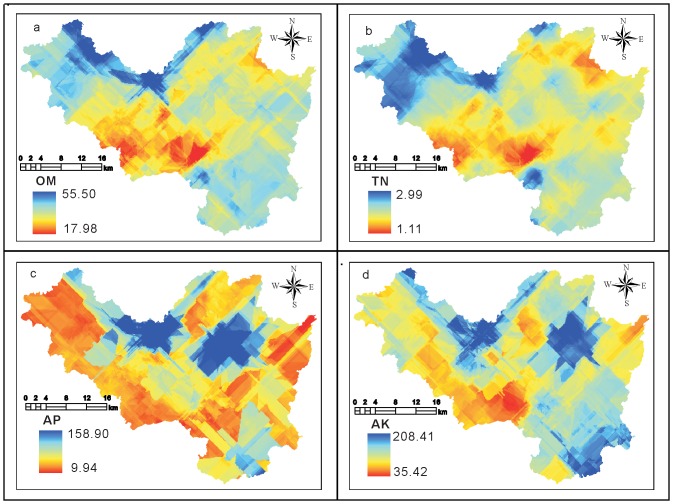
Cokriging prediction results using the PAVs.

In order to compare the above interpolation results, OM, TN, AP, and AK were set as predicted targets for Cokriging analysis, and the three poorest significant correlation variables were selected as auxiliary variables [27], [28]. ES, EMG, and CC were selected as auxiliary parameters for the interpolation of OM. ES, CH, and β were selected as auxiliary parameters for the interpolation of TN. β, CC, and EC were selected as auxiliary parameters for the interpolation of AP. ES, EC, and cosα were selected as auxiliary parameters for the interpolation of AK. The prediction results are presented in Figure 3 [29].

### Accuracy validation

To compare the accuracy of the two methods for the above interpolation, measured test dataset values were compared to the simulation results for the two types of interpolation. The results are presented in Table 3 [30].

**Table 3:** Results of the Interpolation Accuracy.

	Mean		Std. Deviation		Average Absolute Error		Correlation Coefficient with Measured Values	
	BAV	PAV	BAV	PAV	BAV	PAV	BAV	PAV
OM	3.31	3.26	0.54	0.45	0.02	−0.052	0.58	0.292
TN	0.2	0.2	0.03	0.03	−0.002	0.037	0.504	0.446
AP	29.48	29.56	18.38	18.44	7.616	15.619	0.245	0.244
AK	87.48	87.55	25.9	26	12.47	35.343	0.16	0.16

According to the above accuracy comparison results, interpolation using the BAVs and PAVs had similar mean and discrete data. However, results using the BAVs had significantly smaller absolute errors than those using the PAVs in addition to higher correlation coefficients with the measured values. Therefore, the BAV method has higher prediction accuracy than the PAV method [31].

## Conclusions

This article studied the method to select the best auxiliary parameters when performing a Cokriging interpolation of soil nutrient attributes. The most relevant parameters for Cokriging interpolations of soil nutrient attributes were selected by determining the relationship among soil trace elements, terrain attributes, and soil nutrient attributes by exploring the correlation intensity of multi-source data with soil nutrient data based on the correlations among the optional auxiliary parameters. Finally, this article selected the BAVs and PAVs for the Cokriging interpolation results and verified the optimal parameter selection method for Cokriging interpolation used in this paper.

1) The use of auxiliary variables that are more highly correlated leads to higher prediction accuracy. In the process of choosing soil nutrient attributes for Cokriging prediction based on a correlation analysis between the optional auxiliary parameters and interpolation target attributes, optimizing the auxiliary parameter correlation will yield better prediction results.

2) To select auxiliary parameters, a concrete analysis of the stability of auxiliary parameters and suitable conditions should be conducted. As demonstrated by the results obtained here, the influencing factors of the terrain parameters are relatively stable for optional auxiliary parameters. The relationship of the influencing factors of the terrain parameters to nutrient factors is also relatively constant, so the correlations are similar compared with other auxiliary parameters. Therefore, preference should be given to terrain parameters as auxiliary parameters.

3) Data for multiple soil nutrients are typically obtained at the same time. Therefore, it is more convenient to use nutrient data as the auxiliary parameter. Furthermore, soil trace element data may be limited by the experimental conditions or their availability. Therefore, the selection of auxiliary parameters based on different characteristics must consider the data in light of different research needs for auxiliary parameter selection and optimization.

This article only considered soil trace elements, topography, and soil nutrient elements in the selection of optimal auxiliary parameters. Considering this limited range of auxiliary parameters, future research will consider additional optional auxiliary parameters, such as remote-sensing data and soil spectral data, based on expanding the scope of the research data to find better auxiliary variables in order to improve the precision of soil nutrient kriging interpolation.
